# A Cluster of Dengue Cases in Travelers: A Clinical Series from Thailand

**DOI:** 10.3390/tropicalmed6030152

**Published:** 2021-08-14

**Authors:** Hisham A. Imad, Juthamas Phadungsombat, Emi E. Nakayama, Lapakorn Chatapat, Phimphan Pisutsan, Wasin Matsee, Watcharapong Piyaphanee, Wang Nguitragool, Tatsuo Shioda

**Affiliations:** 1 Mahidol Vivax Research Unit, Faculty of Tropical Medicine, Mahidol University, Bangkok 10400, Thailand; wang.ngu@mahidol.edu; 2 Thai Travel Clinic, Hospital for Tropical Diseases, Faculty of Tropical Medicine, Mahidol University, Bangkok 10400, Thailand; lapakorn@thaitravelclinic.com (L.C.); phimphan@thaitravelclinic.com (P.P.); wasin.mat@mahidol.edu (W.M.); watcharapong.piy@mahidol.ac.th (W.P.); 3 Department of Viral Infections, Research Institute for Microbial Diseases, Osaka University, Osaka 565-0871, Japan; juthamas@biken.osaka-u.ac.jp (J.P.); emien@biken.osaka-u.ac.jp (E.E.N.); shioda@biken.osaka-u.ac.jp (T.S.); 4 Department of Clinical Tropical Medicine, Faculty of Tropical Medicine, Mahidol University, Bangkok 10400, Thailand; 5 Department of Molecular Tropical Medicine and Genetics, Faculty of Tropical Medicine, Mahidol University, Bangkok 10400, Thailand

**Keywords:** dengue, *Flavivirus*, serotype-1, primary infection, dengue warning signs

## Abstract

Dengue is an overlooked tropical disease for which billions of people are at risk. The disease, caused by a *Flavivirus* with four distinct serotypes, is transmitted primarily by urban *Aedes* mosquito species. The infection leads to a spectrum of clinical manifestations, with the majority being asymptomatic. Primary dengue fever and, to a greater extent, a subsequent infection with a different serotype is associated with increased severity. Increased global travel and recreational tourism expose individuals naïve to the dengue viruses, the most common arboviral infections among travelers. We describe a cluster of possible primary acute dengue infections in a group of 12 individuals who presented to Bangkok Hospital for Tropical Diseases in 2017. Infection was confirmed by dengue NS1 antigen and multiplex real-time RT-PCR. Nine individuals required hospitalization, and four developed dengue warning signs. Leukocytes, neutrophils, and platelets declined towards defervescence and were negatively correlated with day of illness. Six clinical isolates were identified as dengue serotype-1, with 100% nucleotide identity suggesting that these patients were infected with the same virus.

## 1. Introduction

Dengue remains one of the leading causes of hospitalization in endemic regions [1]. Seventy percent of dengue infections occur in Asia, with annual estimates of over 390 million infections and 96 million cases [2]. Earlier descriptions of dengue reflected the morbidity of the illness. For example, in Africa, fatalities from dengue were described as “ka dinga pepo”, which translates into “devil’s disease” [3]. In Philadelphia, Pennsylvania, USA, dengue was referred to as break-bone fever [4]. Europeans spoke the word dengue during the trans-Atlantic slave trade [5].

The causative agent of dengue is a single-stranded RNA *Flavivirus*, which was first isolated during World War II in Japan and Hawaii [6,7]. Dengue is an arbovirus of great concern to public health, similar to other *Flaviviridae*, such as the yellow fever virus, Japanese encephalitis virus, Zika virus, and West Nile virus [8]. The dengue virus (DENV) has four distinct serotypes (DENV1–4) and two to six genotypes within each serotype. Persons infected with one serotype mount a lifelong immunological response to the same serotype, but only transient protection lasting several months against the remaining serotypes [9]. Serotype-specific severity has been previously described [10,11,12].

The majority of individuals infected with DENV do not develop symptoms and serve as a reservoir of the virus [13,14,15]. Serological surveys reported that 80% of individuals living in endemic regions are seropositive [16]. Humans develop symptoms after an average incubation period of 3 days [17]. These symptoms include: high-grade fever, loss of appetite, headache, gastrointestinal symptoms, myalgia, rash, and bleeding [18]. The clinical course is self-limiting, and symptomatic patients recover within 10 days after the onset of symptoms. Subsequent infections with heterogeneous serotypes are associated with endothelial dysfunction that causes intravascular plasma to leak into the extravascular spaces [19]. This disequilibrium of intravascular volume peaks at defervescence and causes hypotension [20]. Without intervention to correct low blood pressure, the clinical course can rapidly deteriorate, leading to profound shock and multiple organ failure. To date, no approved antiviral agent exists for dengue. Although several candidate vaccines have shown promising results, only one vaccine, Dengvaxia^®^, has been approved for ages 9–45 years. However, use of this vaccine is limited to individuals with a prior dengue infection; it is not recommended for dengue-naïve individuals.

In Thailand, vaccination against dengue is approved, and dengue is holoendemic in the country, with an observed trend of large outbreaks occurring every 4 years. All four serotypes are in circulation at any given time of the year. Bangkok, a metropolitan city located in the tropical region of Thailand, provides habitat for the Asian tiger mosquito (*Aedes albopictus*) and *Aedes aegypti* [21]. Although *Aedes albopictus* is also frequently found in suburban and rural environments, both of these mosquito species are found in urban habitats and breed in close proximity to humans and have heterogeneous biting trends. These behaviors facilitate dengue transmission in an environment without adequate vector control and preventive measures, leading to sporadic cases that are reported between outbreaks. The predominant circulating serotype in Thailand from 2007 to 2017 was DENV-1 [22]. In 2017, there were 6107 cases reported from Bangkok through the end of October, and 53,190 cases were reported by the end of the year in the entire country. As Thailand is a top travel destination due to its diversity of landscapes and cultures, the country attracts millions of travelers annually, and some fall victim to tropical diseases, such as dengue [23,24,25,26,27,28].

Considerable research has focused on secondary dengue infection due to its association with disease severity [12]. Literature on primary dengue infections is limited, especially in regard to reports concerning infections in regions with decreasing dengue-naïve populations. Why some individuals are symptomatic during primary infection while others remain asymptomatic is unknown [29]. Primary symptomatic infections can be severe, leading to hospitalization and sometimes death [30,31,32].

This report examines a cluster outbreak of dengue in a group of travelers (*n* = 12) who visited Bangkok in October 2017 for recreational tourism (training in martial arts, Muay Thai kickboxing). These travelers were from three different provinces of mainland China, Hainan, Guizhou, and Zhejiang. None of the affected individuals had a history of symptomatic dengue infection.

## 2. Materials and Methods

### 2.1. Patient Cohort

We retrospectively reviewed the de-identified clinical data for a group of 12 Chinese travelers who presented to Bangkok Hospital for Tropical Diseases with symptoms of fever during mid-October through mid-November. Dengue infections were diagnosed using a commercially available lateral flow kit (Biosynex, Swiss S.A., Fribourg, Switzerland) that detects the nonstructural protein 1 (NS1) antigen. In addition, six aliquots of 120 μL of serum corresponding to six patients were collected from the available leftover specimens of routine investigations for further serological and molecular analyses. For the remaining six cases, there were no leftover acute-phase specimens available for molecular analysis. The patients reported no travel history outside of Bangkok, and all patients stayed in the dormitories where the recreational activities took place. Some tropical diseases, such as murine typhus and leptospirosis, were not considered in the differential diagnosis due to negative history of exposure to rodents or floods in Bangkok. There was no clinical suspicion of coinfections with other arboviruses circulating in Bangkok or cosmopolitan viruses.

### 2.2. Dengue Detection, Serotyping, and Envelope Nucleotide Sequencing

Dengue antigen testing was performed using a commercially available lateral flow kit that detects the NS1 antigen (Biosynex, Fribourg, Switzerland). For dengue serology, a commercially available kit was used to detect IgM and IgG (SD, Bioline, St. Ingbert, Germany) in accordance with the manufacturer’s protocol. Dengue serotype was determined using a Genesig dengue subtyping multiplex kit, a commercial one-step reverse transcriptase multiplex real-time PCR assay conducted according to the manufacturer’s instructions (Primerdesign, Camberley, UK) using a CFX96TM real-time PCR cycler (Bio-Rad, Hercules, CA, USA). Viral RNA was extracted from serum using a QIAamp Viral RNA Mini Kit and then further amplified using a One-Step RT-PCR Kit (Qiagen, Foster City, USA) with specific primers for position 935–2419 of the envelope region, as published in a previous study [33]. The sequencing reaction was prepared using BigDye Terminator v1.1 (Applied Biosystems, Foster City, USA) and run on an ABI 3130XL sequence analyzer. The obtained sequences were manually aligned to reference sequences of DENV-1 Mochizuki (AB074760) using AliView [34]. The newly generated sequences were deposited in GenBank (accession numbers LC642557–LC642562).

### 2.3. Phylogenetic Analysis

Dengue genotype identification and epidemiological characteristics were analyzed using a phylogenetic method. A nucleotide sequence dataset was prepared, including the newly obtained sequences, NCBI Blast result sequences exhibiting nucleotide sequence identity >99.60%, recent epidemic strains from 2015 to present, and the reference genotype sequence. The sequence dataset was aligned using AliView [34], and a maximum likelihood (ML) tree was constructed in IQ-TREE under TIM2 + F + 4, with 1000 ultrafast bootstrap replicates [35,36]. The ML tree was visualized using FigTree software program.

### 2.4. Data Analysis

Clinical and laboratory data were retrieved anonymously using a standardized case record form. All information was transferred to an electronic data sheet using Microsoft Excel and analyzed using Statistical Package for the Social Sciences (SPSS) software and GraphPad Prism software. Exposure duration was estimated by calculating the number of days from the time of arrival in Bangkok until onset of symptoms. Time of presentation referred to the duration from the onset of symptoms to hospital arrival. The acute phase was defined as the febrile period with a body temperature ≥38.5 °C and presenting to the hospital within 1 to 3 days after the onset of symptoms. Defervescence was estimated based on the time at which oral temperature decreased to below 37.7 °C and the patient remained afebrile. Relative bradycardia was identified when a dissociation of pulse and temperature was observed. A leukocyte count of less than 3500 cells/μL was defined as leukopenia, mild neutropenia when neutrophils were in the range 1000–1500 cells/μL, and severe neutropenia when there were <500 neutrophils/μL. To segregate the patients into groups based on dengue with or without warning signs of severe dengue, we referred to the WHO 2009 dengue clinical classification guideline [37]. These include abdominal pain or tenderness, persistent vomiting, clinical fluid accumulation, mucosal bleed, lethargy or restlessness, liver enlargement, or evidence of hemoconcentration with rapid decline of platelet counts. Further, we compared adolescents (10 to 19 years of age) and adults (20 years and older) by grouping them into Group A = with warning signs and Group B = without warning signs. Distributive frequencies of clinical findings were estimated using all-group analysis. All categorical variables were tested for observed frequencies using Fisher’s exact test. The Wilcoxon and Mann–Whitney tests were used for nonparametric testing. Nonparametric Spearman’s rank correlation coefficients were calculated to explore correlations among the hematological indices, clinical data, and days of illness.

## 3. Results

The patient group included eight male adolescents, three male adults, and one female adult. The median age was 18 years with an interquartile range (IQR) of 16.25–22.25 years. Ten patients became symptomatic within a window of less than 4 weeks of arrival (Figure S1). The median time from the onset of symptoms to presentation to the hospital was 2 days, with an IQR of 1.25–2.75 days. All patients presented during the febrile phase, except for a single patient, who presented at the time of defervescence. No patients exhibited lymphadenopathy. In addition, there was no clinical evidence of plasma leakage, which can occur primarily with secondary dengue infection.

Nine of the 12 patients required hospitalization, and 4 of the 9 hospitalized patients developed dengue warning signs, which included hepatomegaly. The frequency of headache was significantly higher among patients requiring hospitalization (Table S1). The median recorded oral temperature was 39.1 °C (IQR: 38.5–39.5 °C), the median heart rate was 82 beats per minute (IQR: 68.5–95.5 beats per minute), and 83.3% of the patients had relative bradycardia. Other common symptoms among the 12 patients were headache (58%); nausea (42%); myalgia, rash, and loss of appetite (33%); dizziness, vomiting, diarrhea, and bleeding (17%); and abdominal pain and fatigue (8%). No patient in this group reported retro-orbital pain or arthralgia. The median leukocyte count was 3900/μL (IQR: 2250/μL–4800/μL). The median neutrophil, lymphocyte, and platelet counts were 2730/μL, 489/μL, and 173,000/μL, respectively, as shown in Table S2.

At the time of presentation, all 12 cases were positive for dengue NS1, the basis of their dengue diagnosis. However, serology was available only for two cases. A primary-type antibody response (positive IgM and negative IgG) was detected in the first case, and neither anti-IgM nor IgG was detected in the latter case. Molecular multiplexing analysis revealed that six patients were infected with the DENV-1 serotype. The median cycle threshold among these six cases was 19.88 (IQR: 18.86–25.44).

### 3.1. Descriptive Analysis of Warning Signs by Age Group

We did not observe any statistically significant differences in the clinical parameters between adolescents and adults (Table S3). Among the adolescents, the duration of exposure tended to be shorter in the group with warning signs (Group A) compared with the group without warning signs (Group B), with a median of 10 days versus 16 days (*p* = 0.046) (adjusted *p* = 1.000). Similarly, in the adolescents, the body mass index tended to be lower in Group A (*p* = 0.025) (adjusted *p* = 1.000). All adolescents in Group A had relative bradycardia, as did 80% of those in Group B. Among adults, there were no significant differences observed between those who developed warning signs and those who did not (Table 1).

### 3.2. Hematological Profile during Illness

The median hemoglobin level and hematocrit percentage were within normal ranges, and no significant differences were observed during the febrile phase or at defervescence. However, the median leukocyte, neutrophil, and platelet counts tended to decline at defervescence. There were no other differences observed in the hematological profile (Table 2).

### 3.3. Analysis of Correlations between Clinical Parameters

We observed some correlations within the hematological profile in this cohort. Heart rate at presentation was positively correlated with recorded body temperature (*r_s_* = 0.696, *p* = 0.014). A positive correlation was also observed between heart rate and leukocyte count (*r_s_* = 0.606, *p* = 0.039) and neutrophil count (*r_s_* = 0.606, *p* = 0.039). Similarly, the monocyte count was positively correlated with leukocyte count (*r_s_* = 0.711, *p* = 0.0002), neutrophil count (*r_s_* = 0.711, *p* = 0.0002), and lymphocyte count (*r_s_* = 0.510, *p* = 0.015) (Supplementary Material Figures S2 and S3).

**Figure 1:**
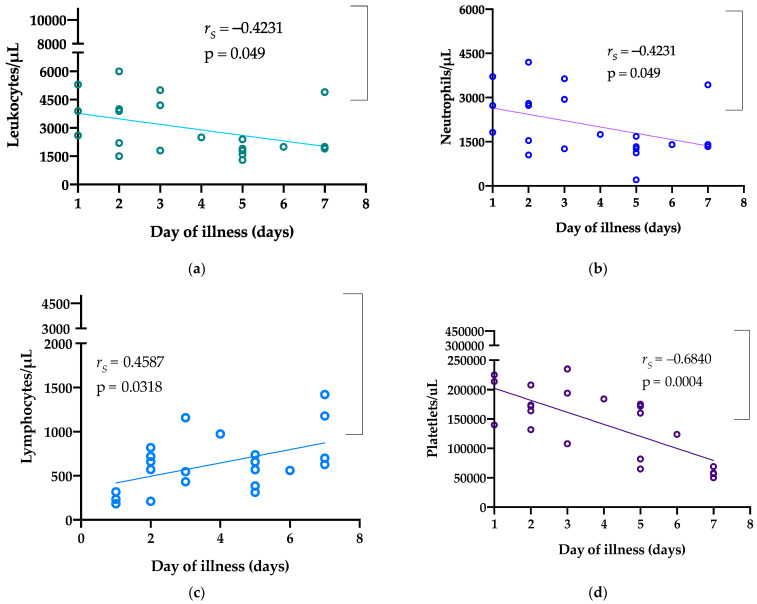

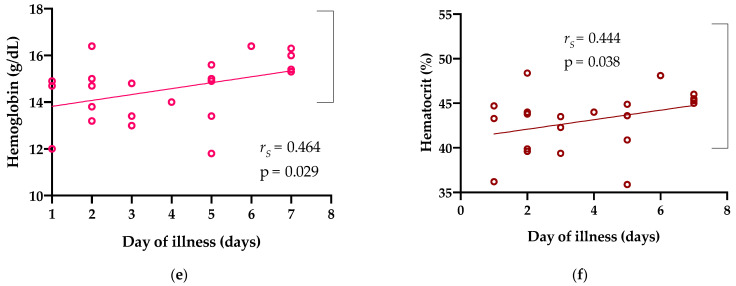
Spearman’s correlation analysis of hematological profile kinetics during the acute phase. Trends observed with leukocytes (**a**), neutrophils (**b**), lymphocytes (**c**), platelets (**d**), and hemoglobin (**e**) and hematocrit (**f**) levels. Normal ranges of parameters are shown in square brackets.

Leukocyte, neutrophil, and platelet counts were negatively correlated with day of illness, whereas lymphocyte count and hemoglobin and hematocrit levels were positively correlated with day of illness (Figure 1).

### 3.4. Phylogenetic Analysis

Samples from six cases were subjected to envelope gene sequencing and demonstrated 100% nucleotide identity. The obtained sequences were searched for similarity to other DENV-1 sequences using the NCBI Blast database. Our sequences had the highest nucleotide identity (99.87%) to a DENV-1 strain from China collected in November 2017 (MT856285.1) and a strain isolated from a traveler returning to China after visiting Thailand in January 2019 (MN923098.1). Our strain shared 99.60–99.80% similarity with China, Hong Kong, and Thailand strains detected in 2018–2019. Myanmar strain 2015 (MG894863) was isolated the earliest among these highly related strains.

**Figure 2:**
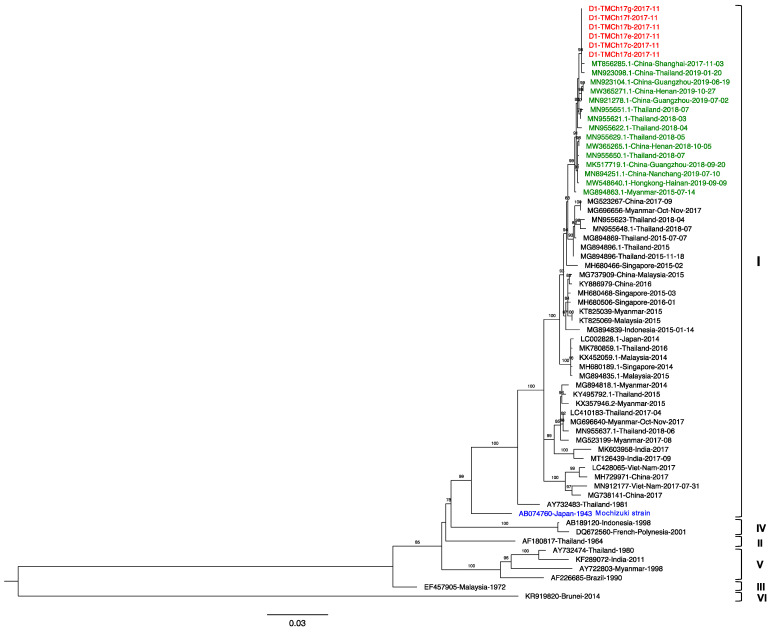
Maximum likelihood tree of DENV-1 based on the 1485 bp envelope region sequence. The tree was constructed using TIM2 + F + 4 with 1000 bootstrap replications. Bootstrap values >75 indicate a relationship to the adjacent branch. Sequences obtained in the present study, related sequences sharing nucleotide identity >99.60%, and the Mochizuki strain are indicated in red, green, and blue, respectively. DENV-1 genotypes are indicated by the brackets to the right.

The phylogenetic tree (Figure 2) revealed that our DENV-1 sequences were genotype I, which was first recognized in Thailand in 1981 and has continuously circulated for over 30 years [38]. Furthermore, this genotype has dynamically circulated all over Southeast Asia for decades [39,40,41]. The specimens were collected in November 2017, which was the early phase of the Thailand DENV-1 wave from 2018 to present. The sequences related to strains including Thailand, China, and Myanmar strains isolated from 2015 to 2017 were consistent with the epidemic reported in this region [42,43]. Moreover, the sequences related to Thailand and China strains were isolated later, in 2018–2019. Although the patients in the present study exhibited symptoms and then recovered while in Thailand, travel-associated transmission must be considered, especially between holoendemic and other endemic areas [44].

## 4. Discussion

Here, we described the clinical manifestations, including blood cell kinetics, during acute dengue infections in a group of travelers in Thailand. All patients arrived in Bangkok via a direct flight from mainland China, and dengue infections were detected at the hospital within a 4-week window period. In estimating the median exposure period, we excluded two patients whose arrival dates were uncertain. As a result, the shortest exposure duration was 4 days, and the longest was 23 days. In previously described studies, seroconversion occurred in 2.9% of 447 travelers after traveling for a month in endemic regions and in 6.7% of 104 travelers after traveling for 6 months [45,46].

Clusters of dengue fever have been defined as two or more cases within a perimeter of 200 m in which the onset of symptoms occurred within 3 weeks [47]. In this cohort, the majority of patients (9/12) became symptomatic within 3 weeks. Some patients were from provinces in China with a history of dengue outbreaks [48]. Six were symptomatic within 2 weeks of arrival, whereas the remaining patients became symptomatic more than 2 weeks after arrival. However, the first two patients who fell ill remained in Thailand for an extended duration beyond 2 weeks and likely had acquired the virus in Bangkok.

An earlier description of primary dengue infections among children living in endemic regions was reported to be mild, and hospitalization was infrequent [14]. Others have reported observations of primary dengue infections in up to 15% of children (median ± SD age: 7.9 ± 2.9 years) and 34% of adults (median ± SD age: 26.6 ± 9.9 years) [49,50]. Previous studies demonstrated that age is associated with symptomatic illness in dengue [29]. Further, viremia was demonstrated to be lower in asymptomatic cases compared with symptomatic cases [15]. All patients in our cases were healthy adolescents and young adults; this age group might be more likely to develop symptoms during dengue infection, although we do not know the total number of travelers who were exposed to dengue in this group. 

In Nicaragua, 36% of children with primary dengue due to DENV-1 infection required hospitalization [51]. Primary dengue infections reportedly occur in as many as 97% of adults from nonendemic regions visiting endemic regions [52]. Although we were unable to determine the sequence of infection in some cases in this cohort, 75% of the patients required hospitalization. The prototype DENV-1 serotype known as the Mochizuki strain later diverged to genotypes I, II, III, IV, and V in different geographic regions [53]. DENV-1 virulence has also been described elsewhere [54]. We suspect that patients infected with the DENV-1 serotype have a higher chance of becoming symptomatic during primary infection.

A striking observation in the present study was that 80% of the patients had relative bradycardia, which was reported in up to 60% of the patients in a previous prospective study [55]. This phenomenon of relative bradycardia, known as Liebermeister’s rule, has been described as occurring in several tropical diseases caused by intracellular, atypical Gram-negative bacteria, parasites, or viruses [56,57,58]. In resource-limited settings, this clinical finding can be useful when considering the differential diagnosis [59]. Furthermore, bradycardia in dengue-infected patients can result from direct virus-associated myocardial injury, which often is benign, transient, and self-limiting [60]. Whether relative bradycardia is associated with primary infection is unknown. In this cohort, there was no difference in having bradycardia with or without warning signs.

Hepatomegaly, one of the seven dengue warning signs, was not exhibited in majority of the adolescents and adults. Liver involvement in dengue is associated with increased severity [61]. In this cohort, the liver enzymes were not analyzed in all cases. However, we observed some mild to moderate elevation of transaminase. Transaminitis in dengue is often transient, due to either direct viral injury of liver cells or irreversible hypoxic insult from shock [62]. Impairment of liver function in dengue infection contributes to the hemorrhagic manifestations of dengue [63,64]. We previously demonstrated that increased expression of interleukin-8 is associated with hepatitis and bleeding in dengue infection [65]. In this cohort, gastrointestinal symptoms were reported in some adolescents but rarely in adults. A predominance of gastrointestinal symptoms after primary dengue infection was reported in a previous dengue cohort in Chinese patients naïve to dengue in Singapore [52]. Diarrhea is a common problem associated with travel and can be misleading as gastroenteritis, potentially leading to an unnecessary course of antimicrobials [66]. Other causes, such as dengue, should be considered when a new onset of diarrhea occurs in conjunction with high-grade fever in tropical regions [67]. Similarly, in this cohort, diarrhea occurred earlier during the illness, overlapping symptoms of gastroenteritis.

Some limitations of this study include not being able to demonstrate primary infections in all cases or identify the serotype in all cases. As all patients promptly presented to the hospital following the onset of symptoms, diagnostics mainly focused on antigen detection, and anti-IgM and IgG levels were only available in two cases, which indicated a primary infection. We were also only able to obtain serum from six patients. In addition, due to the retrospective nature of this report, we were unable to directly interview the patients to obtain information regarding their knowledge, attitudes, and practices regarding dengue and its prevention while visiting endemic regions. We loosely associated age and infecting serotype as playing a role in whether patients become symptomatic during primary infection. Further research is required to understand what factors are in play in asymptomatic dengue patients that could help in the development of new therapeutics against dengue infection.

Advocacy to promote awareness of dengue infection and transmission of the virus will help travelers visiting endemic regions take all the necessary precautionary steps to prevent dengue by preventing mosquito bites. In conclusion, dengue remains a potential problem for travelers visiting endemic regions. In addition, cluster outbreaks can occur in groups traveling together in the absence of adequate precautionary measures.

## Figures and Tables

**Table 1:** Comparison of patients with and without dengue warning signs by age group.

	Adolescents		*p*-Value		Adults		*p*-Value	
	Group A (*n* = 3)	Group B (*n* = 5)		Adjusted *p*-Value	Group A (*n* = 1)	Group B (*n* = 3)		Adjusted *p*-Value
Exposure duration, days	10 (9)	16 (12)	0.046	1.000	8 (8)	20 (4)	0.655	1.000
Body mass index, kg/m^2^	21.0 (17.6)	22.7 (22.0–26.2)	0.025	1.000	23.1 (23.1)	25.5 (22.9)	0.655	1.000
Temperature, °C	39.3 (39.0)	39.0 (38.3–39.5)	0.539	1.000	37.3 (37.3)	39.2 (38.4)	0.180	1.000
Heart rate, beats per minute	82.0 (82.0)	82.0 (60.0–98.5)	0.878	1.000	68 (68)	80 (70)	0.180	1.000
Mean arterial pressure, mmHg	73.3 (68.0)	79.3 (73.0–85.0)	0.101	1.000	70.3 (70.3)	97 (90.6)	0.180	1.000
Day of presentation, days	2 (2)	2 (1.5–2.5)	0.491	1.000	5 (5)	1 (1)	0.157	1.000
Hospitalization, *n* (%)	3 (100)	3 (60)	0.464	1.000	1 (100)	2 (66.7)	0.505	1.000
Fever, *n* (%)	3 (100)	5 (100)	NA	NA	1 (100)	3 (100)	NA	NA
Dizziness, *n* (%)	0	2 (40)	0.464	1.000	0	0	NA	NA
Relative bradycardia, *n* (%)	3 (100)	4 (80)	1.000	1.000	0	3 (100)	0.250	1.000
Hepatomegaly, *n* (%)	1 (33.3)	0	0.375	1.000	1 (100)	0	0.250	1.000
Headache, *n* (%)	2 (66.7)	3 (60)	1.000	1.000	0	2 (66.7)	1.000	1.000
Myalgia, *n* (%)	1 (33.3)	1 (20)	1.000	1.000	0	2 (66.7)	1.000	1.000
Arthralgia, *n* (%)	0	0	NA	NA	0	0	NA	NA
Rash, *n* (%)	0	2 (40)	0.464	1.000	1 (100)	1 (33.3)	1.000	1.000
Bleeding, *n* (%)	1 (33.3)	0	0.375	1.000	1 (100)	0	0.250	1.000
Loss of appetite, *n* (%)	2 (66.7)	1 (20)	0.464	1.000	1 (100)	0	0.250	1.000
Nausea, *n* (%)	2 (66.7)	1 (20)	0.464	1.000	1 (100)	1 (33.3)	1.000	1.000
Vomiting, *n* (%)	1 (33.3)	1 (20)	1.000	1.000	0	0	NA	NA
Abdominal pain, *n* (%)	1 (33.3)	0	0.375	1.000	0	0	NA	NA
Diarrhea, *n* (%)	1 (33.3)	1 (20)	1.000	1.000	0	0	NA	NA
Fatigue, *n* (%)	0	1 (20)	1.000	1.000	0	0	NA	NA

Data are presented as median (interquartile range) and actual numbers (percent). In some, only the median and the 25th percentile are provided due to low number of cases. Day of presentation represents the day the patient visited the hospital after developing symptoms. Group A refers to cases with warning signs. Group B refers to cases without warning signs.

**Table 2:** Hematological profile during the febrile and defervescent phases of dengue infection.

Parameters	Febrile Phase	Defervescence	*p*-Value	Adjusted *p*-Value
Hemoglobin, g/dL (14.00–18.00)	14.70 (13.20–14.90)	15.15 (13.85–15.70)	0.121	1.000
Hematocrit, % (40.00–54.00)	43.30 (39.60–44.00)	44.45 (42.93–45.35)	0.130	1.000
Leukocytes/µL (4000–11,000)	3,900 (1900–3900)	1900 (1750–2100)	0.010	0.090
Neutrophils/µL (2500–6000)	2730 (1540–3640)	1330 (1225–1470)	0.010	0.090
Lymphocytes/µL (1000–4800)	546 (234–720)	642 (516–797)	0.260	1.000
Monocytes/µL (300–900)	156 (75–265)	130 (60–209)	0.231	1.000
Eosinophils/µL (50–500)	0 (0–26)	26 (0–66)	0.138	1.000
Basophils/µL (0–300)	216 (136–420)	292 (210–402)	0.481	1.000
Platelets/µL (150,000–450,000)	174,000 (140,000–214,000)	103,000 (63,250–172,750)	0.015	0.135

The data are provided as median (interquartile range). Normal ranges of parameters are shown in square brackets.

## Data Availability

The data presented in this study are available on request from the corresponding author. The data are not publicly available to ensure the privacy of the study participants.

## Supplementary Materials

The following are available online at https://www.mdpi.com/article/10.3390/tropicalmed6030152/s1, Figure S1: Exposure duration in endemic region. Figure S2: Analysis of correlations between heart rate and temperature, leukocyte count, and neutrophil count. Figure S3: Correlations between monocyte count and other white blood cell indices. Table S1: Descriptive analysis of the patient cohort. Table S2: Descriptive analysis of cases involving dengue warning signs. Table S3: Descriptive analysis by age group.
