# Supplementary material for: A Cluster of Dengue Cases in Travelers: A Clinical Series from Thailand
Source: Trop Med Infect Dis. 2021 Aug 14;6(3):152. doi: 10.3390/tropicalmed6030152 (PMC8396219; doi:10.3390/tropicalmed6030152)
Supplement: Supplementary file 1 [file tropicalmed-06-00152-s001.zip › tropicalmed-1336467-SI.pdf]

Tropical Medicine and Infectious Disease

MDPI

Supplementary Materials

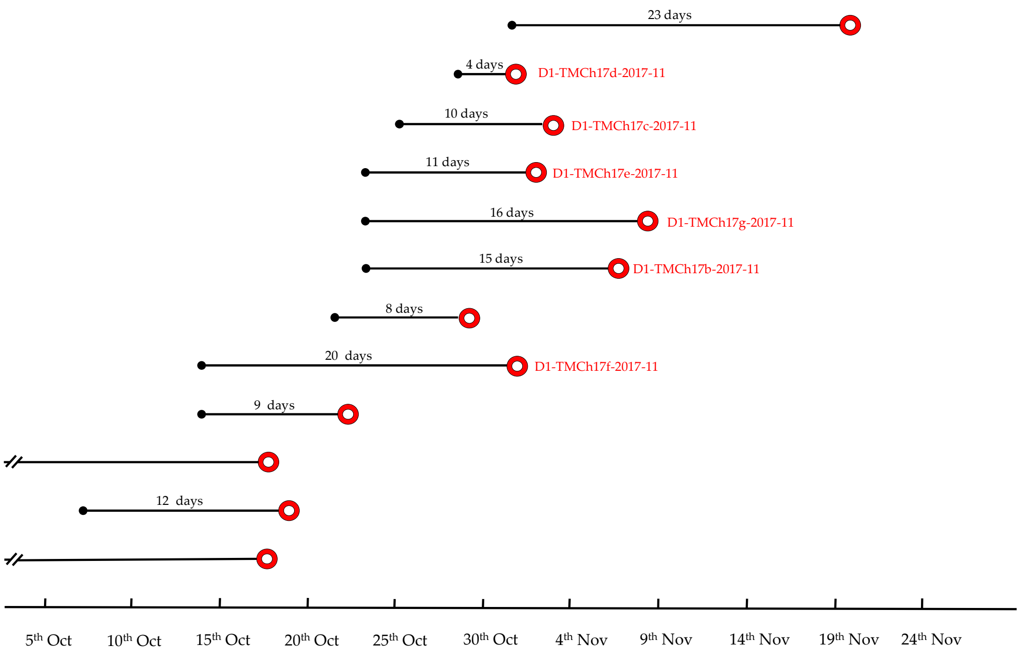

Figure S1. Time from date of arrival in Bangkok until symptoms developed, indicated in red with the sequence reference number.

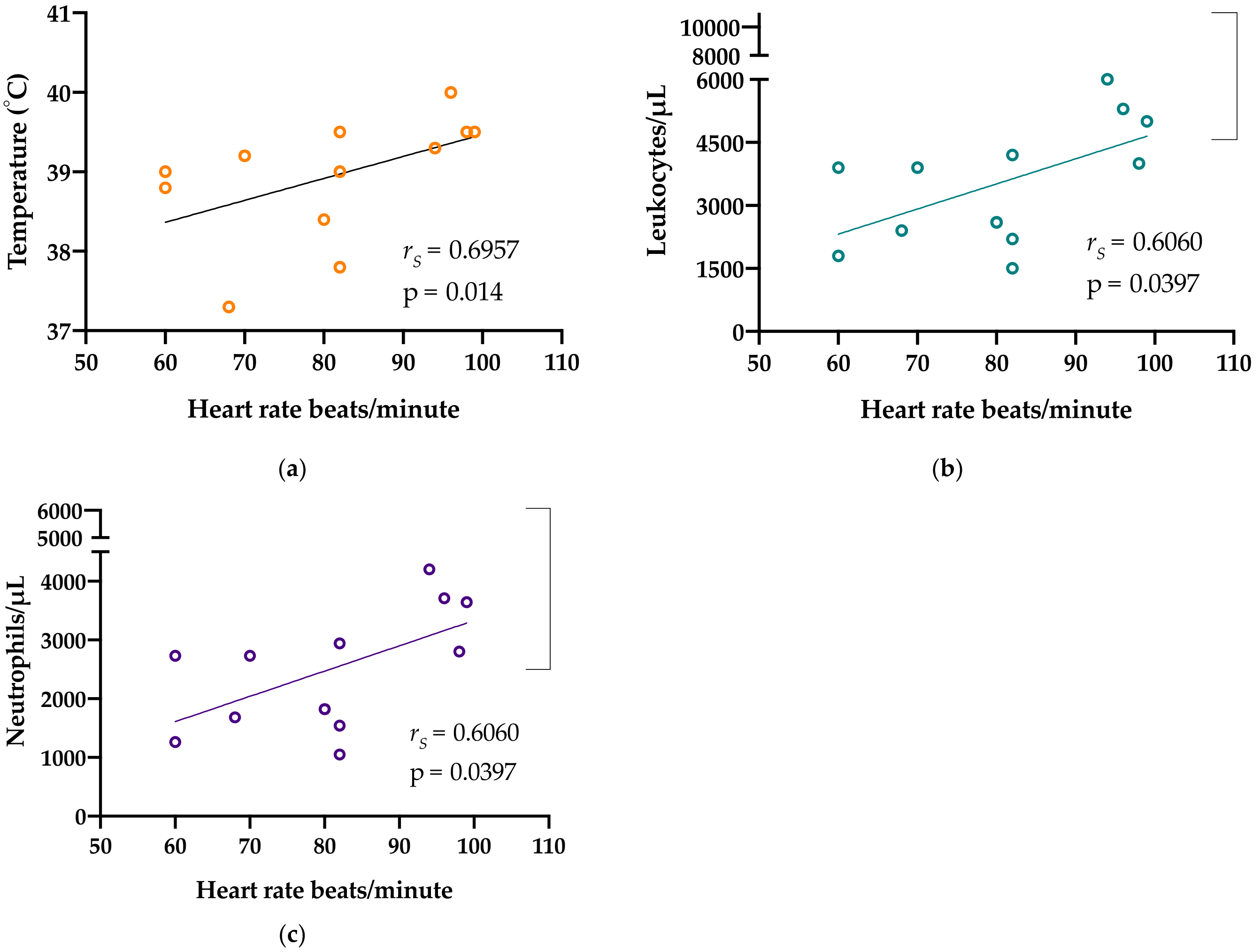

Figure S2. Spearman's correlation analysis of heart rate and temperature, leukocyte count, and neutrophil count. (**a**) Temperature and heart rate; (**b**) Leukocyte count and heart rate; (**c**) Neutrophils and heart rate. Normal ranges of parameters are shown in square brackets.

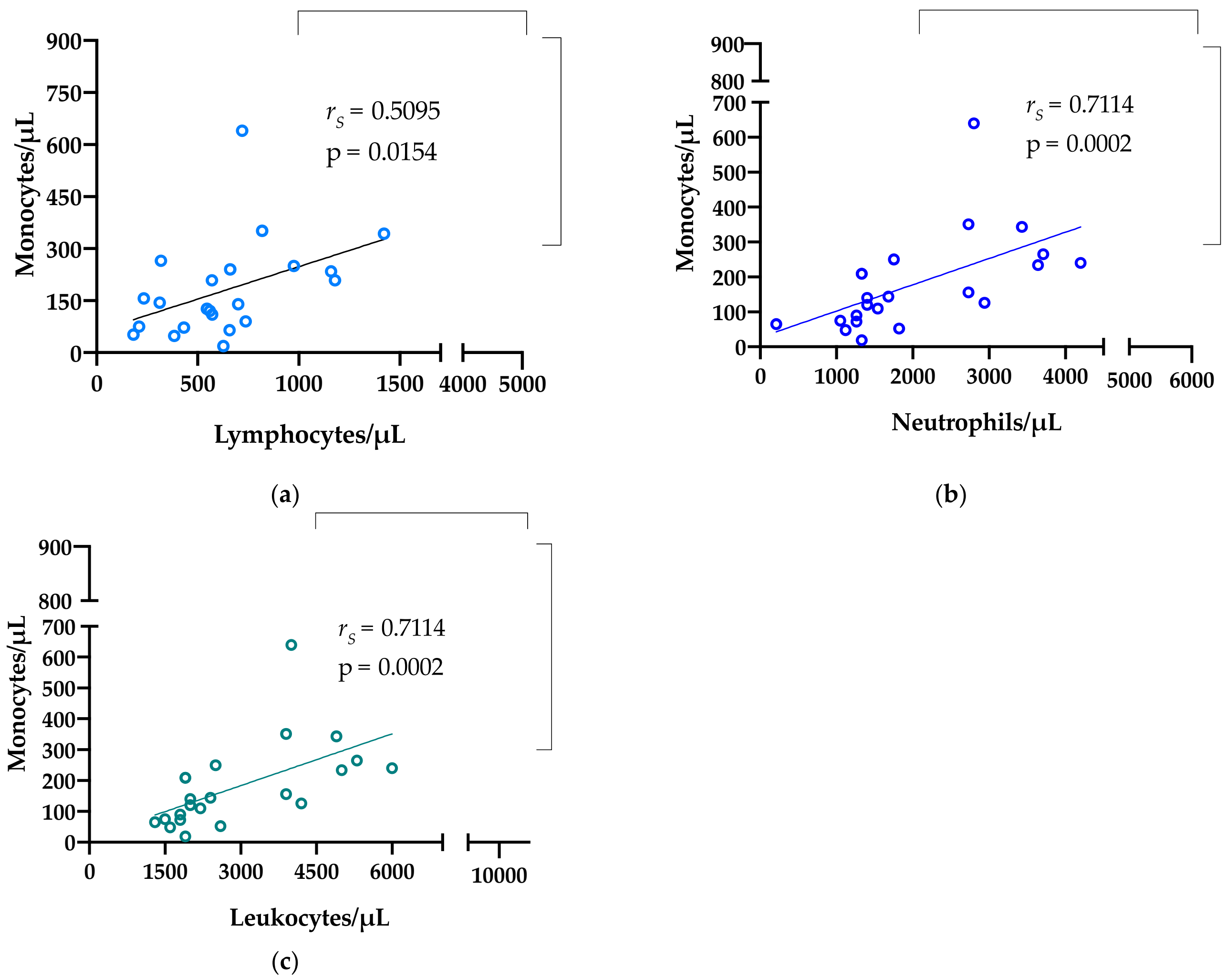

Figure S3. Spearman's correlation of monocyte count with other white blood indices (**a**) Monocytes with lymphocytes; (**b**) Monocytes with neutrophils; (**c**) Monocytes with leukocytes. Normal ranges of parameters are shown in square brackets.

Table S1. Descriptive analysis of the patient cohort.

	Dengue infection (12)	Inpatient (9)	Outpatient (3)	p value	adjusted p value
Exposure duration, days	11.5 (8.7–17.0)	13.50 (9.25–19.0)	8 (4)	0.295	1.000
Age, years	18.0 (16.2–22.2)	17.0 (15.5–21.5)	19 (16)	0.576	1.000
Body mass index kg/m^2	22.7 (21.2–25.2)	21.9 (21.0–24.1)	24.68 (22.60)	0.229	1.000
Temperature °C	39.1 (38.5–39.5)	39.3 (38.7–39.5)	38.80 (37.80)	0.225	1.000
Hear rate, beats per minute	82.0 (68.5–95.5)	82.0 (74.0–97.0)	70 (60.00)	0.192	1.000
Mean arterial pressure, mmHg	77.1 (71.5–90.0)	74.6 (70.6–89.3)	82.00 (79.33)	0.166	1.000
Day of presentation, days	2.0 (1.2–2.7)	2.0 (1.5–3.0)	2 (1)	0.370	1.000
Dizziness	2 (16.7)	2 (22.2)	0	1.000	1.000
Relative bradycardia	10 (83.3)	8 (88.9)	2 (66.7)	0.455	1.000
Hepatomegaly	2 (16.7)	2 (22.2)	0	1.000	1.000
Headache	7 (58.3)	7 (77.8)	0	0.045	1.000
Myalgia	4 (33.3)	3 (33.3)	1 (33.3)	1.000	1.000
Rash	4 (33.3)	4 (44.4)	0	0.491	1.000
Bleeding	2 (16.7)	2 (22.2)	0	1.000	1.000
Loss of appetite	4 (33.3)	4 (44.4)	0	0.491	1.000
Nausea	5 (41.7)	5 (55.6)	0	0.205	1.000
Vomiting	2 (16.7)	1 (11.1)	1 (33.3)	0.455	1.000
Abdominal pain	1 (8.3)	1 (11.1)	0	1.000	1.000
Diarrhea	2 (16.7)	2 (22.2)	0	1.000	1.000
Fatigue	1 (8.3)	1 (11.1)	0	1.000	1.000
Hemoglobin, g/dL [14.00–18.00]	14.70 (13.25–14.97)	14.70 (13.30–14.95)	13.80 (13.00)	0.781	1.000
Hematocrit, % [40.00–54.00]	43.40 (39.67–43.95)	43.50 (40.95–44.35)	39.90 (39.40)	0.309	1.000
Leukocytes /µL [4500–11,000]	3900 (2250–4800)	3900 (2400–4800)	2400 (1500)	0.308	1.000
Neutrophils /µL [2500–6000]	2730 (1575–3465)	2730 (1680–3675)	1680 (1050)	0.308	1.000
Lymphocytes /µL [1000–4800]	489 (253–705)	572 (276–769)	312 (210)	0.229	1.000
Monocytes /µL [300–900]	150 (83–258)	234 (91–308)	126 (75)	0.309	1.000
Eosinophils /µL [50–500]	0 (0–46)	0 (0–39.50)	0 (0–0)	0.613	1.000
Basophils /µL [0–300]	68 (137–417)	216 (138.45–426)	408 (120)	0.926	1.000
Platelets /µL [150,000–450,000]	173000 (134000–212500)	174000 (136000–219500)	164000 (82000)	0.309	1.000
AST /IUL [0–40]	36.50 (31.50–45.25)	33 (22.50–40.50)	40 (39)	0.101	1.000
ALT /IUL [0–41]	16.50 (13.25–59.00)	17 (11–45)	16 (14)	0.653	1.000
Ct value [15–30]	19.88 (18.86–25.44)	19.95 (18.04–25.62)	19.82 (19.82)	0.770	1.000

The data are in median (IQR), including frequencies with percentages. In some variables, only the median and the 25th percentile are provided due to low number of cases. Inpatient refers to cases hospitalized and outpatient refers to cases not requiring hospitalization. Day of presentation represents the day the patient visited the hospital after developing symptoms. Normal ranges of parameters are shown in square brackets.

Table S2. Descriptive analysis of cases with dengue warning signs.

	Warning signs (4)	No warning signs (8)	p value	adjusted p value
Exposure duration, days	9.5 (8.2–10.7)	16.0 (10.0–20.7)	0.087	1.000
Age, years	15.5 (11.0–24.5)	19.0 (17.0–22.2)	0.230	1.000
Body mass index kg/m^2	21.1 (18.4–22.6)	23.7 (22.6–27.1)	0.042	1.000
Temperature °C	39.1 (37.7–39.4)	39.1 (38.5–39.5)	0.797	1.000
Hear rate, beats per minute	82.0 (71.0–91.0)	81.0 (62.0–97.0)	1.000	1.000
Mean arterial pressure, mmHg	71.8 (68.5–74.3)	85.0 (76.0–95.4)	0.017	1.000
Day of presentation, days	2.5 (2.0–4.5)	2.0 (1.0–2.0)	0.082	1.000
Hospitalization, n (%)	4 (100)	5 (62.5)	0.491	1.000
Dizziness	0	2 (25)	0.515	1.000
Relative bradycardia	3 (75)	7 (87.5)	1.000	1.000
Hepatomegaly	2 (50)	0	0.091	1.000
Headache	2 (50)	5 (62.5)	1.000	1.000
Myalgia	1 (25)	3 (37.5)	1.000	1.000
Rash	1 (25)	3 (37.5)	1.000	1.000
Bleeding	2 (50)	0	0.091	1.000
Loss of appetite	3 (75)	1 (12.5)	0.067	1.000
Nausea	3 (75)	1 (12.5)	0.222	1.000
Vomiting	1 (25)	1 (12.5)	1.000	1.000
Abdominal pain	1 (25)	0	0.333	1.000
Diarrhea	1 (25)	1 (12.5)	1.000	1.000
Fatigue	0	1 (12.5)	1.000	1.000
Hemoglobin, g/dL [14.00–18.00]	14.05 (12.35–14.92)	14.75 (13.35–15.42)	0.444	1.000
Hematocrit, % [40.00–54.00]	43.05 (37.72–43.95)	43.40 (39.67–44.42)	0.865	1.000
Leukocytes /µL [4500–11,000]	3050 (1900–3900)	4100 (2450–5225)	0.173	1.000
Neutrophils /µL [2500–6000]	2135 (1330–2730)	2870 (1715–3692)	0.173	1.000
Lymphocytes /µL [1000–4800]	502 (283–757)	432 (235–705)	0.734	1.000
Monocytes /µL [300–900]	133 (81–302)	189 (87–258)	0.734	1.000
Eosinophils /µL [50–500]	0 (0–43)	0 (0–46)	0.762	1.000
Basophils /µL [0–300]	303 (156–444)	266 (132–417)	0.734	1.000
Platelets /µL [150,000–450,000]	191000 (124500–212500)	168000 (134000–217250)	0.734	1.000
AST /IUL [0–40]	30.5 (14)	36.5 (32.5–154.25)	0.739	1.000
ALT /IUL [0–41]	41 (9)	16.5 (13.75–97)	0.737	1.000
Ct value [15–30]	25.62 (25.62)	19.75 (17.22–19.92)	0.064	1.000

The data are in median (IQR), including frequencies with percentages. In some variables, only the median and the 25th percentile are provided due to low number of cases. Day of presentation represents the day the patient visited the hospital after developing symptoms. Normal ranges of parameters are shown in square brackets.

Table S3. Descriptive analysis by age group.

	Adolescent (8)	Adults (4)	p value	adjusted p value
Exposure duration, days	11.5 (9.7–16.0)	14.0 (52.0–2.2)	1.000	1.000
Age, years	17.0 (14.5–18.5)	25.0 (20.7–30.7)	0.006	0.198
Body mass index kg/m^2	21.9 (21.0–24.1)	24.3 (22.9–29.5)	0.062	1.000
Temperature °C	39.1 (38.8–39.5)	38.8 (37.5–39.8)	0.607	1.000
Hear rate, beats per minute	82.0 (65.5–97.0)	75.0 (68.5–92.0)	0.493	1.000
Mean arterial pressure, mmHg	74.8 (71.5–81.3)	93.8 (75.4–104.7)	0.126	1.000
Day of presentation, days	2.0 (2.0–2.7)	1.5 (1.0–4.2)	0.522	1.000
Hospitalization, n (%)	4 (100)	5 (62.5)	1.000	1.000
Dizziness	2 (25)	0	0.515	1.000
Relative bradycardia	7 (87.5)	3 (75)	1.000	1.000
Hepatomegaly	1 (12.5)	1 (25)	1.000	1.000
Headache	5 (62.5)	2 (50)	1.000	1.000
Myalgia	2 (25)	2 (50)	0.547	1.000
Rash	2 (25)	2 (50)	0.547	1.000
Bleeding	1 (12.5)	1 (25)	1.000	1.000
Loss of appetite	3 (37.5)	1 (25)	1.000	1.000
Nausea	3 (37.5)	2 (50)	1.000	1.000
Vomiting	2 (25)	0	0.515	1.000
Abdominal pain	1 (12.5)	0	1.000	1.000
Diarrhea	2 (25)	0	0.515	1.000
Fatigue	1 (12.5)	0	1.000	1.000
Hemoglobin, g/dL [14.00–18.00]	14.75 (13.25–14.97)	14.25 (12.45–15.97)	0.799	1.000
Hematocrit, % [40.00–54.00]	43.55 (40.27–43.95)	41.60 (37.12–47.12)	0.610	1.000
Leukocytes /µL [4500–11,000]	3250 (2500–4800)	4100 (2450–5225)	0.932	1.000
Neutrophils /µL [2500–6000]	2275 (1575–3465)	2870 (1715–3692)	0.932	1.000
Lymphocytes /µL [1000–4800]	559 (342–779)	432 (235–705)	0.308	1.000
Monocytes /µL [300–900]	135 (81.50–223.50)	189 (87–258)	0.308	1.000
Eosinophils /µL [50–500]	0 (0–50.37)	0 (0–46)	0.613	1.000
Basophils /µL [0–300]	312 (137.47–451.50)	266 (132–417)	0.396	1.000
Platelets /µL [150,000–450,000]	157000 (114000–204500)	168000 (134000–217250)	0.396	1.000
AST /IUL [0–40]	39 (32.50–272)	36.5 (32.5–154.25)	0.297	1.000
ALT /IUL [0–41]	17 (16.5–205)	16.5 (13.75–97)	0.024	0.792
Ct value [15–30]	22.61 (17.29–25.80)	19.75 (19.68)	0.355	1.000

The data are in median (IQR), including frequencies with percentages. In some variables, only the median and the 25th percentile are provided due to low number of cases. Day of presentation represents the day the patient visited the hospital after developing symptoms. Normal ranges of parameters are shown in square brackets.
